# Editorial: Impact of radiotherapy and radiosurgery on neuro-oncology

**DOI:** 10.3389/fonc.2022.978709

**Published:** 2022-07-26

**Authors:** Alfredo Conti

**Affiliations:** Neurosurgery, IRCCS Istituto delle Scienze Neurologiche di Bologna, Italy, Dipartimento di Scienze Biomediche e NeuroMotorie (DIBINEM), Alma Mater Studiorum University of Bologna, Bologna, Italy

**Keywords:** radiotherapy, radiosurgery, brain tumors, radiomics, lower grade gliomas

Radiotherapy is one of the main pillars of neuro-oncology. Used universally in all malignant neoplasms affecting the central nervous system, it is increasingly adopted as an alternative to surgical treatment in numerous malignant and benign lesions including brain metastases (BM), meningiomas, vestibular schwannomas, pituitary adenomas or paragangliomas. The clinical settings in which a radiotherapy treatment is indispensable are, in fact, innumerable and at least in three different scenarios: as a preoperative (or neoadjuvant) treatment, as a postoperative (or adjuvant) treatment, and as a rescue therapy. Furthermore, in many circumstances, radiation therapy is used as a primary or upfront treatment as an alternative to surgical management. This broadening of the indications in the use of ionizing radiation in neuro-oncology has been made possible by the astonishing technological evolution that has been witnessed in the last two decades together with the availability of clinical results that have definitively demonstrated the efficacy of radiation treatments, also allowing a progressive refinement of the techniques and methods of treatment. The technological leap obtained by modern radiotherapy allowed a significant increase treatment of efficacy while simultaneously reducing toxicity. To list just a few of them: 3D treatment planning, the extension of the principles of stereotaxis with the possibility of delivering treatments with very efficient dose gradients providing excellent normal tissue sparing, and the evolution of image-guidance. Furthermore, the use of single and multiple fraction radiosurgery to treat brain lesions has gained momentum over the last few years, and turns out to be now more promising than ever (1–3).

If much has been done with great success and with the transition of radiotherapy treatments for neuro-oncology from simple palliation to a fundamental treatment with prospects for healing the disease, many other aspects remain open. Nonetheless, we are in a historical phase in which many opportunities to improve the efficacy of radiotherapy treatments are within reach, above all through both a further improvement of irradiation techniques and a better understanding of the biomolecular characteristics of tumors, of the genetic factors associated with the response to ionizing radiation, and the use of increasingly advanced neuro-imaging modalities that make it possible to identify tumor areas with specific biological characteristics differently responding to treatment. These innovations represent a challenge, which we should investigate in order to ensure that all professionals involved in the care of brain cancer are exposed to cutting-edge developments, in order to keep delivering the best possible treatments.

With this in mind, we have decided to propose this Research Topic of Frontiers in Oncology having as topic the impact of radiotherapy and radiosurgery on neuro-oncology. Among several submissions, we selected 9 high quality contributions providing cutting-edge research in the field, exploring major aspects of radiotherapy for neuro-oncology. Particularly, in this Research Topic, we included one study on frameless robotic radiosurgery reporting one of the largest series on the treatment of residual, recurrent, or metastatic intracranial hemangiopericytomas (solitary fibrous tumor) using Cyberknife. Huang et al. reported data of 28 of these rare tumors, with a control rate of 78.5%, that is higher than the mean of the previous studies. A second study on radiosurgery was authored by Li et al. who analyzed the outcome of gamma knife radiosurgery (GKRS) in an outstanding number (369) of nonfunctioning pituitary adenomas (NFPA) reporting progression free survivals of 100%, 98%, 97%, 86% and 77% at 1, 3, 5, 10, and 15 years, respectively. The authors also identified predictors of tumor control including parasellar invasion and tumor margin dose (<12 Gy).

Two studies provide cutting-edge research in the field of biomolecular characterization of brain tumors applied to radiosensitivity. Li et al. analyzed expression of long non-coding RNAs (lncRNA) to predict the radiotherapy response of lower-grade gliomas. These RNA species belong to a class of non-coding RNA with a length of not more than 200 nucleotides and usually lack coding potential. Several studies have confirmed that lncRNA expression is associated with tumor initiation, progression, and treatment. lncRNAs can modulate tumor radiosensitivity by functioning as competitive endogenous RNA (ceRNA). In this study, for the first time, authors systematically investigated the mechanism of ceRNA regulation in the radiosensitivity of LGG based on RNA-seq data and database predictions. In the second study, Du et al. developed a radiosensitivity prediction model based on hypoxia genes for LGG by using weighted correlation network analysis (WGCNA) and least absolute shrinkage and selection operator (Lasso). 12 genes (AGK, ETV4, PARD6A, PTP4A2, RIOK3, SIGMAR1, SLC34A2, SMURF1, STK33, TCEAL1, TFPI, and UROS) were included in the model. A radiosensitivity-related risk score model was established based on the overall rate of The Cancer Genome Atlas (TCGA) dataset in patients who received radiotherapy.

The development of prediction nomograms based on radiomics analysis was proposed in other two studies. Zhou et al. developed and validated an individualized prognostic nomogram by integrating physiological, volumetric, clinical chemistry, and molecular biological surrogates for predicting survival in patients with brain metastases after stereotactic radiosurgery. The nomogram demonstrated precise risk-stratifications to guide personalized treatment for brain metastases. Zhai et al. developed and validated a radiomics nomogram based on the multiparametric MRI imaging. The radiomics nomogram demonstrated a favorable predictive accuracy of meningioma consistency before surgery, which showing the potential of clinical application. Finally, three studies propose some hot-topics in radiotherapy field (4, 5), including new techniques in fractionated photon and proton radiotherapy.

Ahn et al. describe a series of 123 patients who underwent hippocampal-avoidance whole-brain radiation therapy (HA-WBRT) and analyzed the risk of BM in the hippocampal areas using multi-variable logistic regression, classification and regression tree (CART) analyses, and gradient boosting method (GBM).

Eichkorn et al. report results in terms of effectiveness and toxicity of fractionated proton beam radiotherapy for treatment of cranial nerve schwannomas unsuitable for photon stereotactic radiosurgery. Treatment resulted in a promising 100% local control with cranial nerve functional protection rate of 80%.

Arpa et al. propose an innovative approach to improve the efficacy of hypofractionated stereotactic radiotherapy (HSRT) after hyperbaric oxygen therapy (HBO) for the treatment of recurrent high-grade glioma (rHGG) and herein report the results of an ad interim analysis. Results are promising and could represent an alternative, with low toxicity, to systemic therapies.

In conclusion, we are proud of the results obtained with this Research Topic which provides an innovative look at many of the fundamental themes of radiotherapy in the neuro-oncology field. The proposed studies are all state-of-the-art and realistically represent a foundation for future research on the topic.

## Author Contributions

The author confirms being the sole contributor of this work and has approved it for publication.

## Conflict of Interest

The author declares that the research was conducted in the absence of any commercial or financial relationships that could be construed as a potential conflict of interest.

## Publisher’s Note

All claims expressed in this article are solely those of the authors and do not necessarily represent those of their affiliated organizations, or those of the publisher, the editors and the reviewers. Any product that may be evaluated in this article, or claim that may be made by its manufacturer, is not guaranteed or endorsed by the publisher.

## References

[1] Conti A Acker G Kluge A Loebel F Kreimeier A Budach V. The role of minimally invasive treatment modalities. Front Oncol (2019) 9:915. doi: 10.3389/fonc.2019.00915 31608228 PMC6761912

[2] Conti A Pontoriero A Salamone I Siragusa C Midili F La Torre D. Protecting venous structures during radiosurgery for parasagittal meningiomas. Neurosurg Focus (2009) 27(5):E11. doi: 10.3171/2009.8.FOCUS09-157 19877789

[3] De Maria L Terzi di Bergamo L Conti A Hayashi K Pinzi V Murai T. CyberKnife for recurrent malignant gliomas: A systematic review and meta-analysis. Front Oncol (2021) 11:652646. doi: 10.3389/fonc.2021.652646 33854978 PMC8039376

[4] Lambrecht M Eekers DBP Alapetite C Burnet NG Calugaru V Coremans IEM. Radiation dose constraints for organs at risk in neuro-oncology; the European particle therapy network consensus. Radiother Oncol (2018) 128(1):26–36. doi: 10.1016/j.radonc.2018.05.001. work package 1 of the taskforce “European Particle Therapy Network” of ESTRO. 29779919

[5] Fernández E Morillo V Salvador M Santafé A Beato I Rodríguez M. Hyperbaric oxygen and radiation therapy: A review. Clin Transl Oncol (2021) 23(6):1047–53. doi: 10.1007/s12094-020-02513-5 33206332

